# Are the Kids Alright? Dehydration and High Temperatures During Pregnancy Impact Offspring Physiology, Morphology, and Survival in a Cold-adapted Lizard

**DOI:** 10.1093/icb/icaf082

**Published:** 2025-06-09

**Authors:** George A Brusch, Jean-François Le Galliard, Robin Viton, Rodrigo S B Gavira, Jean Clobert, Olivier Lourdais

**Affiliations:** Centre d'Etudes Biologiques de Chizé, CNRS, 79360Villiers en Bois, France; Department of Biological Sciences, California State University San Marcos, San Marcos, CA 92096, USA; Sorbonne Université, CNRS, IRD, INRAe, Institut d’écologie et des sciences de l'environnement (IEES), 4 Place Jussieu, 75252 Paris, Cedex 5, France; Ecole normale supérieure, Département de biologie, CNRS, UMS 3194, Centre de recherche en écologie expérimentale et prédictive (CEREEP-Ecotron IleDeFrance), PSL University, 11 chemin de Busseau, 77140, Saint-Pierre-lès-Nemours, France; Centre d'Etudes Biologiques de Chizé, CNRS, 79360Villiers en Bois, France; Centre d'Etudes Biologiques de Chizé, CNRS, 79360Villiers en Bois, France; Station d'Ecologie Théorique et Expérimentale de Moulis, CNRS, UMR 5321, Saint Girons, France; Centre d'Etudes Biologiques de Chizé, CNRS, 79360Villiers en Bois, France; School of Life Sciences, Arizona State University, Tempe, AZ 85287-4501, USA

## Abstract

Climate change will continue to increase mean global temperatures, with daily minima increasing more than daily maxima temperatures on average. In addition, altered rainfall patterns due to climate change will disrupt water availability. Such changes are likely to influence thermo-hydroregulation and reproduction strategies in terrestrial ectotherms. We manipulated access to preferred diurnal temperature (9 h vs. 4 h at preferred temperature), nocturnal temperature at rest (22 vs. 17°C) as well as water availability during gestation (± *ad libitum* access to water) in female common lizards (*Zootoca vivipara*), a cold- and wet-adapted species. We previously reported that hot conditions (day and night) accelerated gestation but high nighttime temperatures increased the burden on females already constrained by heavy resource and water investment during gestation. We expanded the understanding of this relationship by examining the effects of maternal hydration and temperature on offspring (neonates and juveniles; *N* = 625) physiology (water loss rates and respiratory activity), morphology, performance (endurance capacity and growth), and survival. On average, longer access to preferred temperature during the day conferred benefits on offspring growth and survival, despite a negative effect on body condition at birth. High nighttime temperatures during gestation reduced offspring postnatal growth during early life and, together with high daytime temperatures, reduced tail width and endurance capacity at birth as well as offspring survival. Additionally, water deprivation poses a challenge to homeostasis, but offspring demonstrate resilience in coping with this potential stressor and these effects were not stronger in hot climates. Notably, the benefits of hotter environments are not always additive, highlighting the complexity of temperature-mediated effects on maternal and offspring outcomes.

## Introduction

Maintaining homeostasis requires coordinated regulation of individual state variables, for example, body temperature or whole-body hydration, and organisms living in fluctuating environments must therefore allocate limited resources and time to essential regulatory processes such as thermoregulation and hydroregulation (Bernhardt et al. 2020). Trade-offs between these regulatory processes may be important drivers of the plasticity and evolution of life history strategies, which describe how fast animals grow and reproduce and how long they live (Stearns 1998; Rozen Rechel et al. 2019; Dupoué et al. 2020). During particularly resource-intensive life-history stages such as reproduction, an organism’s ability to balance these essential functions is crucial, but a stable internal environment can be difficult to maintain. This is challenged by resource demands of the developing progeny that combine energy requirements (Koteja 2000), temperature regulation (Farmer 2003), and water allocation (Zani and Stein 2018). In this context, fluctuations in environmental (abiotic) conditions can impose a significant burden by altering maternal physiology and developmental trajectories (Brusch et al. 2023). Understanding how organisms respond when faced with multiple, concurrent changes in the environment is essential to understand how compounded challenges may disrupt physiological processes and ultimately threaten reproductive success (Jackson et al. 2021). This is particularly relevant in the face of climatic changes that affect both diurnal and nocturnal temperature (Rutshmann et al. 2024) and also desiccation risks (Riddell et al. 2019).

The majority of animals are ectotherms and are therefore reliant on environmental conditions for thermoregulation, which influences their metabolic rates, activity levels, and overall physiological functions (Tewksbury et al. 2008). Their response to global climate change is therefore especially relevant given their ability to maintain stable internal body temperatures may be challenged by shifting environments. External temperatures may significantly impact ectotherms during reproduction by influencing their reproductive timing, success rates, and embryonic development. For instance, higher temperatures can accelerate development but may also lead to increased stress or mortality, while cooler temperatures can delay reproduction and alter offspring quality or survival (Le Henanff et al. 2013; Lorioux et al. 2013). Furthermore, the considerable resource requirements during reproduction can lead to resource trade-offs between mothers and offspring (i.e., intergenerational trade-offs). Historically, the currency of interest in studies of intergenerational trade-offs has been energy; however, recent research has shifted towards consideration of other fundamental resources, because non-energetic resources like water are crucial to reproductive success in terrestrial ectotherms (Dupoué et al. 2015; Dupoué et al. 2018; Brusch et al. 2020; Wayne et al. 2025). This is also especially important considering that climate change impacts both temperature and precipitation patterns. Thus, together, external temperatures and resource availability impact ectotherms during reproduction by affecting their behaviors, reproductive timing, and success. Access to preferred temperatures can enhance mating activities and increase offspring viability, while extreme temperatures can lead to stress or reduced reproductive output, and limited resources can hinder ectotherms ability to meet the requirements needed for reproduction or parental care (Rutshmann et al. 2024).

Heretofore, many studies have examined ectothermic responses to a warming climate, with the vast majority focusing on increasing daily mean temperatures (Huey and Tewksbury 2009; Díaz et al. 2022; Doucette et al. 2023). However, there has been a recent focus on ectothermic responses to nocturnal warming, which is outpacing diurnal warming in most regions, as well as to concurrent shifts in rainfall patterns (Rutschmann et al. 2021, 2024; Brusch et al. 2023). It is still largely unclear how diurnal ectotherms will be affected by independent shifts in daytime and nighttime temperatures. Rising temperatures during the day should first modify thermoregulation (increased access to preferred temperature) but ultimately result in deviation above critical limits. In turn, rising temperatures at night may influence performance or increased energetic costs. Reproduction is a particularly sensitive period that requires heavy water allocation to embryos during gestation and significant metabolic costs throughout pregnancy (Pincheira‐Donoso et al. 2013). Viviparous species, like the common lizard (*Zootoca vivipara*), may be more susceptible to the effects of climate change during reproduction because of the substantial maternal investment during pregnancy (Foucart et al. 2014; Dupoué et al. 2018). Viviparity in terrestrial ectotherms may also generate intergenerational trade-offs for energetic and hydric resources, and these physiological trade-offs may be further altered by climate change.

Previously, we independently manipulated temperature during the daytime and nighttime as well as water availability (± *ad libitum* access to water) throughout pregnancy in female *Z. vivipara*, a cold- and wet-adapted species. Using climatic chambers, we mimicked either long (9 h) or short (4 h) access to preferred temperature during the day. At the same time, we mimicked warm (22°C) or cold (17°C) shelters at night, and manipulated water availability (dry or wet). Our temperature and water treatments were meant to mimic conditions that lizards are already facing at the warm margin of their distribution. Common lizards are facing local extinction at the southern border of their distribution as climate change leads to hotter and drier conditions. These lizards thrive in moist environments, and the increasing temperatures and reduced rainfall are diminishing their preferred habitats (Rutschmann et al. 2024). We found that elevated temperatures during the day or night shortened gestation periods and heightened the resource demands on mothers. Additionally, hot days combined with water restrictions resulted in maternal dehydration. While water restrictions did not seem to influence reproductive output, they did affect a mother’s capacity to allocate resources. Finally, hot days positively impacted reproductive output, whereas hot nights had a detrimental effect (Brusch et al. 2023).

Herein, we expand the understanding of these reproductive trade-offs by examining transgenerational effects of female hydration and temperature availability on offspring physiology, morphology, performance, and survival. Our aim is to test the hypothesis that intergenerational trade-offs over limited energetic and hydric resources lead to morphological and physiological impacts on neonates. Our general hypothesis is that accessing preferred temperature (daytime) is critical for development but that warm nocturnal conditions and water constraints can negatively impact reproduction are this cold-adapted species. Specifically, we examined the following predictions:

1. We first predicted that water deprivation during gestation alters female investment into her offspring and thus impacts neonate traits (body mass and size) and physiology (heart and breathing rate, trans-epidermal water loss) with potential carry-over effects on postnatal growth and survival of neonates.
2. Second, we predicted that an extended access to preferred temperature should be beneficial by advancing breeding phenology in warmer environments if neonate fitness also depends on timing of birth (“the sooner the better hypothesis,” see Shine and Olsson 2003; Le Henanff et al. 2013).
3. Third, we predicted that compounded high temperatures (e.g., hot days and hot nights) during gestation would negatively impact neonate quality and fitness, especially their survival, and especially hot nighttime conditions that challenged the energy balance in gravid females.

## Materials and methods

### Study species and housing

Adult *Z. vivipara* are small-bodied lizards (snout-vent length, SVL 50–75 mm) typically found in cool mesic habitats (e.g., peat bogs) and have a wide geographic range across northern Eurasia with both oviparous and viviparous reproductive modes (Dely and Böhme 1984). This species is an active thermoregulator and females maintain temperature close to 31°C during pregnancy. Reproductive females used for this study were captured in May and June 2019 (*n* = 135) from populations of the viviparous reproductive mode at the Plateau de Millevaches (Limousin) and transported to the Centre d'Etudes Biologiques de Chizé, France (CEBC). We confirmed that all females captured were in the end stages of vitellogenesis using ultrasonography (Sonosite MicroMaxx, Bothell, WA) and previously described methods (Gilman and Wolf 2007). They were held in captivity in standard conditions during an acclimation period of at least seven days until June 2019, when they started gestation. For the majority of pregnancy, daytime and nighttime temperatures were then manipulated using climatic chambers (Vötsch VP 600, Balingen, Germany) where conditions were classified as either “hot day” (9 h at T*_set_*: 31°C) or “cold day” (4 h at T*_set_*: 31°C) and “hot night” (overnight at 22°C) or “cold night” (overnight at 17°C). Water vapor deficits were kept constant among the temperature regimes and females were provided *ad libitum* access to food (crickets). Lizards were further distributed into either “wet” (*ad libitum* access to water) or “dry” (water restricted) conditions for a total of eight different treatment groups (see Brusch et al. 2023 for full protocol and detailed description of maintenance conditions and measurements during gestation).

### Neonates morphology and functional traits

On the day of parturition, offspring were collected and classified as living or stillborn. Living offspring from each litter were placed in 1 L plastic containers containing a moist paper towel and held at 20°C for 6–12 h. We followed a standard procedure to measure morphology, heart and breathing rates, water loss rates, and endurance capacity of neonates at birth.

Morphology and sex identification: each living neonate (*N* = 625) was weighed (± 1 mg). We also collected an image of the ventral surface at 600 dpi using a flatbed scanner (Canon Inc., Canoscan LiDE 400) to measure the SVL and tail width at the 7th sub-caudal scale using ImageJ2 (Rueden et al. 2017) to assess tail condition, which is an essential site for energy storage (Avery 1974; Brusch et al 2018). We performed scale counts to determine neonate sex (Braña 2008). We next marked offspring by toe clipping so that we could later identify surviving neonates in the outdoor enclosures (see below).

Heart rate, breathing rate, and water loss: neonates were allowed to acclimate undisturbed at 20°C for 1 h. After which we used ultrasonography (Sonosite MicroMaxx, Bothell, WA) to measure heart rate within 90 s of removing them from their containers and before any notable increase in heart rates due to handling. We allowed neonates to re-acclimate at 20°C for 1 h before using a stereo microscope (STEMI 508, Zeiss) to measure breathing rate. Marked neonates were placed in individual 50 mL Falcon tubes at 20°C for 12 h without access to water. After 12 h without water, neonates were weighed (± 1 mg) to record their body mass loss, which provides an indirect measure of total evaporative water loss (TEWL), as all neonates lost mass and none had any signs of secreting nitrogenous wastes during this period. Neonates were then provided access to water, and acclimated to ∼25°C for 1–2 h prior to measuring their endurance capacity (Le Galliard et al. 2004).

Stamina: After acclimation, we recorded the room temperature (± 0.1°C), and placed each neonate on a circular, closed track with a circumference of 1.73 m. The arena was place in a temperature-controlled room. We used a soft-bristled paintbrush to gently tap the dorsal surface of neonates to elicit running and removed neonates from the trial after ten continuous taps. We recorded the total distance (cm) and total time (s) that each neonate ran.

### Outdoor enclosures

After all data were collected, we released all living neonates (*n* = 623) into one of ten different outdoor enclosures located near the CEBC premises so that they were equally distributed based on maternal temperature and water treatments. Each enclosure (4 × 4 m) was equipped with artificial shelters, water was provided *ad libitum*, and the vegetation provided a mosaic of basking sites and shelters (Le Henanff et al. 2013). While neonates were in the enclosures, we provided crickets every week as supplemental food to other insects naturally found in the enclosures. Neonates were kept in the enclosures for 49–90 days (depending on parturition date), after which all surviving neonates were captured via pitfalls over a 72 h period. We identified neonates via toe clips, weighed each (± 1 mg), and collected another ventral image using a flatbed scanner (see above) to measure tail width and SVL. Once these measurements were completed, neonates were eventually released at the site where their mothers were captured in natural populations the previous spring. Procedures were performed in accordance with laws relative to the capture, transport, and experimental use of *Z. vivipara* (DREAL permit #13,016_19,042,018) and approved by an independent ethical committee (Apafis #2018060111033048_v5).

### Statistical analyses

We analyzed maternal treatment effects on neonate morphology (SVL, relative tail width [individual tail width—mean tail width of all neonates], and body-condition index [BCI, residuals from a linear regression of neonate mass against SVL]) and each functional trait at birth (heart and ventilation rate, water loss, and endurance) independently using model selection procedures in R software (R Development Core Team, version 4.1.1) with the packages *MASS* (Venables and Ripley 2013), *multcomp* (Hothorn et al. 2008), *car* (Fox and Weisberg 2019), and *lmtest* (Zeileis and Hothorn 2002). Our initial, full models included additive and interactive effects of the three maternal experimental treatments (day- and nighttime temperature and water availability) and were fitted with linear mixed-effects models in the *nlme* package (Pinheiro et al. 2021). We included a random maternal effect to control for non-independence among offspring from the same litter and to compute variance for inter-familial differences. All models also included a sex effect to control for potential differences between males and females. We then used stepwise removal of insignificant coefficients as recommended for null hypothesis testing in manipulative studies focusing on main and interactive effects of treatments (Arnold 2010; Zuur et al. 2010).

Besides sexual differences at birth, each model included relevant covariates based on our *a priori* knowledge of the variability of offspring traits at birth from earlier studies. For our model comparing differences in tail width, we used SVL as a covariate. To model functional traits, we included body mass (for log-transformed TEWL to ensure normality) or BCI for heart and breathing rate. We also log-transformed distance ran data and included room temperature, body size, and speed (distance time^−1^) as covariates because speed could not be perfectly controlled for on our track. A Tukey’s HSD *post hoc* test was used to compare treatment groups. We checked all initial models to ensure the data met the assumptions for parametric testing and used transformations where necessary. Data are presented as mean ± SEM and differences were accepted as significant at the level of *P* < 0.05.

For unknown reasons (e.g., intrusion of a predator, unintentional emigration), no neonates from two of the outdoor enclosures survived. We therefore did not include these enclosures in our analyses. To compare differences in neonate survival and growth, we used a mixed model approach to account for the additive effects of enclosure and maternal identity. Our *a priori* model for survival included effects of treatments (see above) and sex, and SVL and BCI at birth as covariates (assuming positive effects on survival). Exploratory analyses with binomial generalized additive models suggested that effects of covariates were linear, if any, so we did not include non-linear effects. In addition, analyses of neonate survival with maximum-likelihood approaches were not satisfactory because full models did not converge properly and some parameter estimates were not reliable. We therefore decided to fit a logistic regression with a Bayesian approach using *MCMCglmm* (Hadfield 2010). We used the categorical family distribution with a logit link to fit the binary survival data and further used non-informative priors and a fixed residual variance since the model is Bernoulli logistic regression, for which we cannot evaluate residual variance (Nakagawa and Schielzeth 2010). Priors for random components were taken from the Χ2 distribution, which should result in a better inference for binary traits (de Villemereuil et al. 2013), using a long burn-in phase and a thinning interval of >50 results in satisfactory MCMC chains with a sufficient sample size for the calculation of posterior distributions. Posteriors were then examined and we calculated MCMC *P*-values defined as twice the posterior probability that the estimate is negative or positive, whichever is smallest (Hadfield 2010). Because lizards were all different ages when they were removed from the outdoor enclosures, we included birth date as a covariate for our analyses of neonate survival. However, when we included both maternal treatment and birth date as explanatory variables, treatment was significant but birth date was not, and was therefore not included in our final model. We measured growth in terms of mass change or tail width from release until recapture divided by neonate age at recapture (assuming growth is linear). To analyze growth from birth to recapture, we used a linear mixed-effect model with a 3-way treatment interaction, as well as sex, and standardized BCI and birthdate as covariates (i.e., centering and scaling). Stepwise removal of insignificant variables was performed as described above for traits at birth.

## Results

### Neonate morphology at birth

**Fig. 1:**
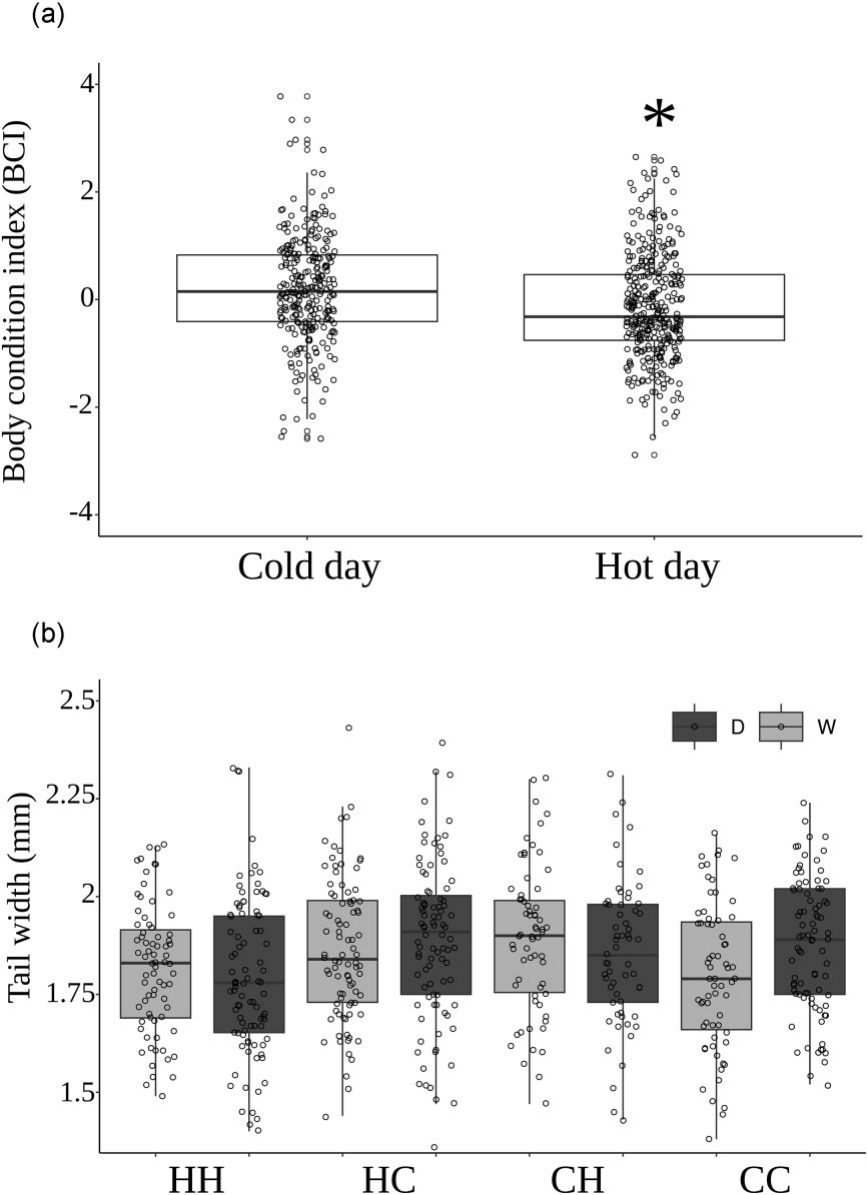
Median and quantile distributions of (a) body condition (BCI) at birth and (b) tail widths at birth of neonate *Z. vivipara* from mothers in wet (W) or dry (D) conditions and exposed to hot day and night (HH), hot day and cold night (HC), cold day and hot night (CH), or cold day and night (CC) throughout the majority of pregnancy. Asterix indicates a significant difference among groups (Tukey’s HSD *post hoc* test). Boxplots of the distribution of data are displayed, whereas circles represent individual neonates.

We did not detect any treatment effects or sex differences of neonate SVL at birth (all *P* > 0.05). However, after accounting for neonate sex (F_1,117_ = 11.30, *P* = 0.008), we found a significant main effect of daytime temperatures on the BCI of neonates (F_1,117_ = 19.85, *P* < 0.001; Fig. 1a). Overall, neonates from mothers exposed to hot days had a lower BCI (−0.15 ± 0.05) compared to those exposed to cold days (0.19 ± 0.06) and female neonates in our study were born with a lower BCI (−0.05 ± 0.01) compared to males (0.03 ± 0.01). Tail width at birth was related to neonate SVL (F_1,113_ = 8.69, *P* = 0.003) and sex (F_1,113_ = 7.07, *P* = 0.008) but it was also influenced by two-way interactions between day and night temperatures (F_1,113_ = 9.58, *P* = 0.002). Neonates from mothers exposed to hot day- and nighttime temperatures had the narrowest tails (1.80 ± 0.01 mm) compared to those exposed to hot days and cold nights (1.88 ± 0.01 mm) or cold days and hot nights (1.87 ± 0.02 mm), while neonates from mothers exposed to cold day- and nighttime temperatures had intermediate tail widths (1.84 ± 0.01 mm). We also found that neonate tail width was influenced by an interaction between night temperatures and water availability (F_1,113_ = 5.44, *P* = 0.020; Fig. 1b), where neonates from mothers in dry conditions had narrower tails when nights were hot (1.82 ± 0.02 mm) or cold (1.82 ± 0.02 mm), and wider tails when conditions were wet during hot (1.85 ± 0.02 mm) or cold nights (1.85 ± 0.02 mm).

### Neonate functional traits at birth

**Fig. 2:**
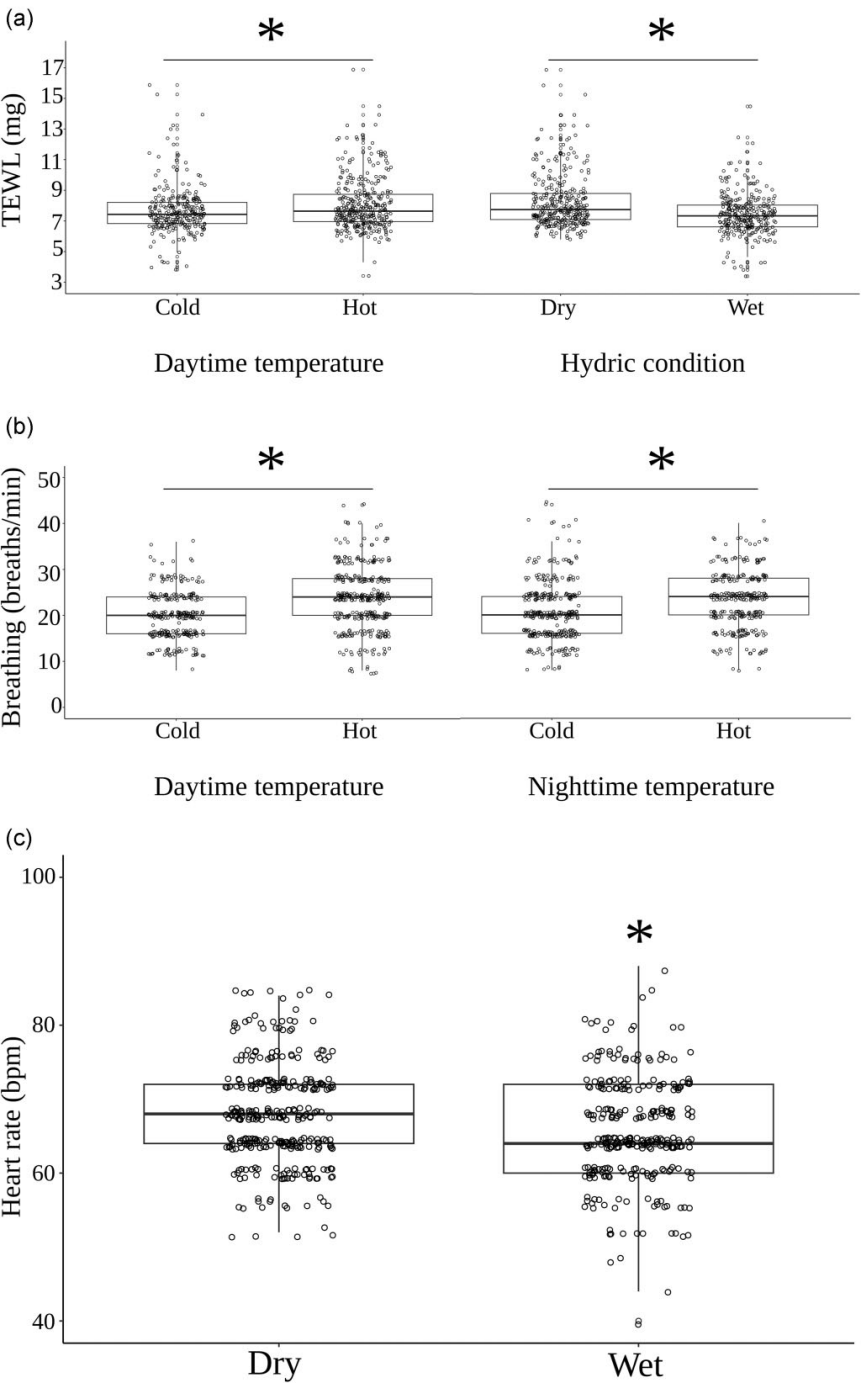
Median and quantile distributions of (a) total evaporative water loss (TEWL, mg per 12 h), (b) breathing rate, and (c) heart rate of neonate *Z. vivipara* from mothers held with (wet) or without (dry) *ad libitum* access to water and exposed to hot day and night (HH), hot day and cold night (HC), cold day and hot night (CH), or cold day and night (CC) throughout the majority of pregnancy. Asterix indicates a significant difference among groups (Tukey’s HSD *post hoc* test). Boxplots of the distribution of data are displayed, whereas circles represent individual neonates.

TEWL at birth was significantly influenced by water availability (F_1,116_ = 46.95, *P* < 0.001) and daytime temperatures during gestation (F_1,116_ = 11.43, *P* = 0.001). Neonates from mothers who were held in dry conditions had significantly higher TEWL rates (8.27 ± 0.10 mg per 12 h) compared to those held in wet conditions (7.44 ± 0.08 mg per 12 h). Additionally, neonates from mothers held in hot days had higher TEWL rates (8.06 ± 0.09 mg) compared to those held in cold days (7.65 ± 0.09 mg; Fig. 2a).

After accounting for a positive effect of BCI (F_1,116_ = 6.52, *P* = 0.011), we found a significant main effect of daytime (F_1,116_ = 15.21, *P* < 0.001) and nighttime temperatures during gestation (F_1,116_ = 6.13, *P* = 0.015) on breathing rates. Neonates from mothers exposed to hot days had higher breathings rates compared to mothers exposed to cold days (23.6 ± 0.4 breaths min^−1^, 19.9 ± 0.3 breaths min^−1^, respectively). Similarly, neonates from mothers exposed to hot nights had higher breathings rates compared to those from mothers exposed to cold nights (23.0 ± 0.4 breaths min^−1^, 21.0 ± 0.4 breaths min^−1^, respectively; Fig. 2b). After also accounting for a positive effect of BCI (F_1,117_ = 4.35, *P* = 0.037), we found a significant main effect of water availability (F_1,117_ = 6.19, *P* = 0.014) on neonate heart rate, where neonates from mothers held without water had higher heart rates (67.9 ± 0.37 bpm; Fig. 2c) compared to neonates from mothers with water (65.6 ± 0.41 bpm).

**Fig. 3:**
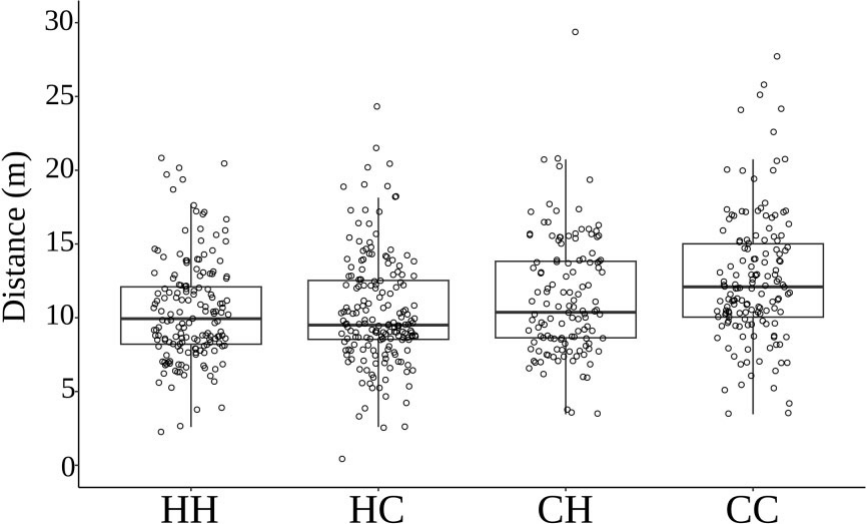
Median and quantile distributions of distance ran by neonate *Z. vivipara* from mothers exposed to hot day and night (HH), hot day and cold night (HC), cold day and hot night (CH), or cold day and night (CC) throughout the majority of pregnancy. Boxplots of the distribution of data are displayed, whereas circles represent individual neonates.

When comparing distance traveled and accounting for positive effects of BCI (F_1,115_ = 51.41, *P* < 0.001) and negative effects of speed (F_1,115_ = 25.55, *P* < 0.001), we found a significant interaction between day and nighttime temperatures on endurance capacity (F_1,115_ = 5.93, *P* = 0.016). Where either hot temperature in the day or night led to lower distance (10.4 ± 0.28 m, 11.4 ± 0.36 m, respectively) ran compared to cold day and night temperatures (12.6 ± 0.35 m) but the effect was not additive when temperatures were hot in the day and night (10.4 ± 0.27 m; Fig. 3).

### Postnatal survival and growth

**Fig. 4:**
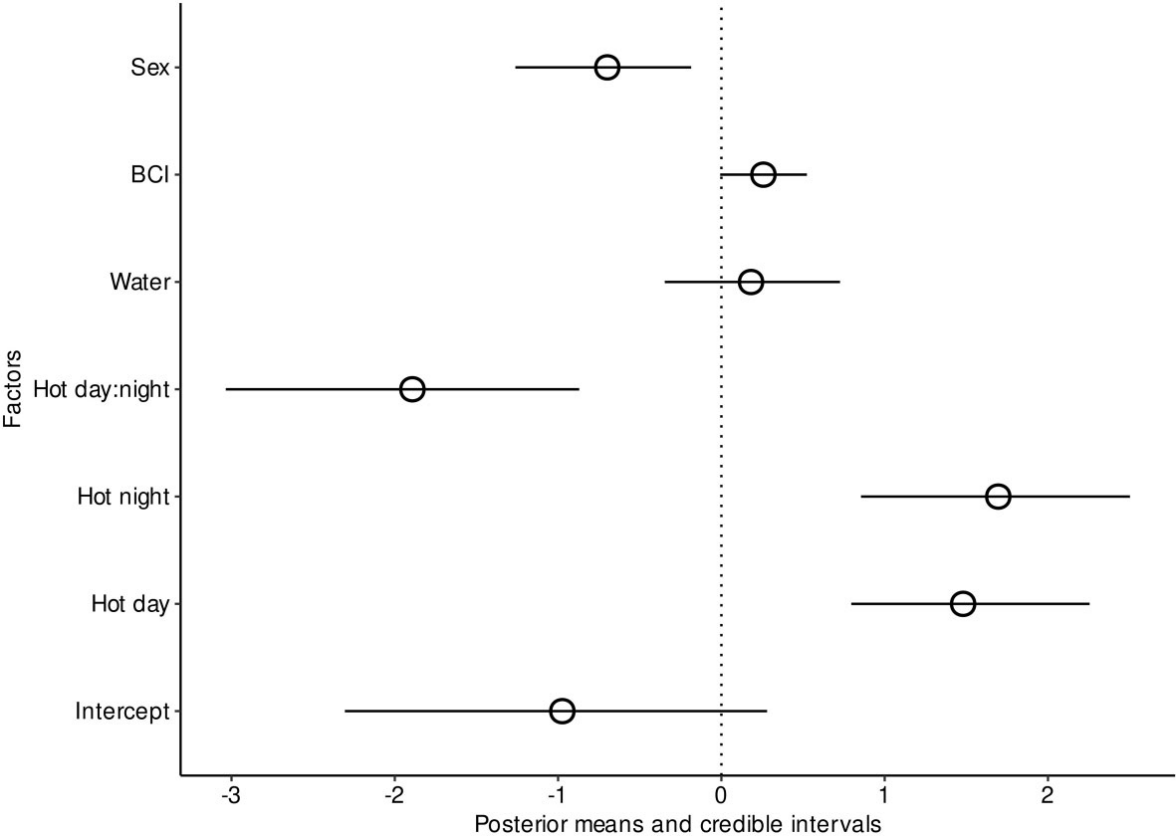
Caterpillar plot of posterior estimates from the Bayesian analysis of juvenile survival probability in outdoor enclosures after the manipulation. Figure provides the posterior mean and credible intervals from a model testing effects of body condition at birth, offspring sex, and temperature treatments.

**Fig. 5:**
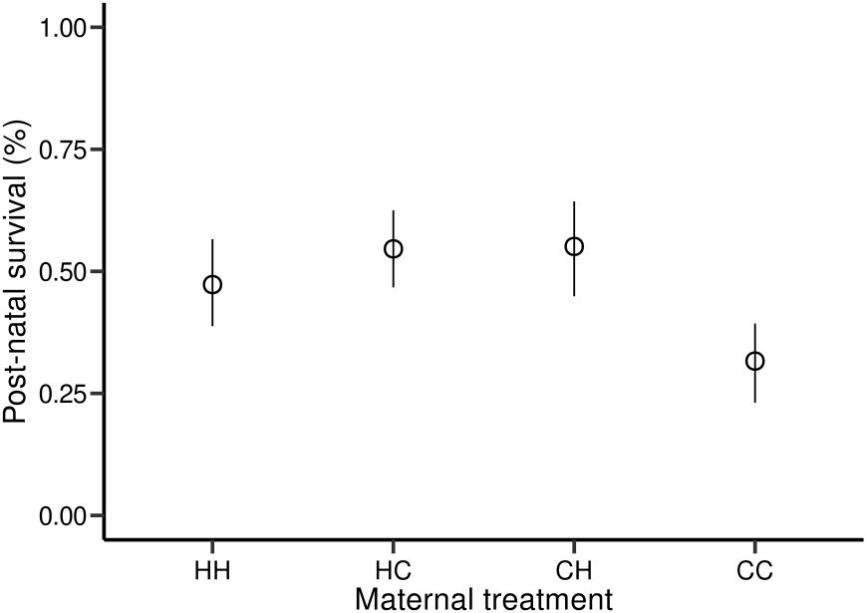
Bootstrap mean and confidence limits of survival percentages of neonate *Z. vivipara* in outdoor enclosures after the manipulation. Offspring were from mothers exposed to hot day and night (HH), hot day and cold night (HC), cold day and hot night (CH), or cold day and night (CC) throughout most of the pregnancy duration.

The best model from our Bayesian analyses of postnatal survival had a significant interaction between day and night temperatures (MCMC *P*-values, *P*-MCMC = 0.001) after accounting for the relationship with BCI (*P*-MCMC = 0.054) and sex differences (*P*-MCMC = 0.014; Fig. 4). Juveniles from mothers exposed to hot days and hot nights were less likely to survive, as were males or those who started in a worse body condition upon release into the enclosures (Fig. 5). When comparing body mass gain of juveniles from release to recapture, there was a negative effect of birth date (F_1,223_ = 215.77, *P* < 0.001) and positive effect of BCI (F_1,223_ = 4.96, *P* = 0.014), and males grew slower than females (F_1,223_ = 5.94, *P* = 0.016). Furthermore, higher nighttime temperatures had a negative effect on mass gain (F_1,223_ = 8.41, *P* = 0.004). Tail width increase was similarly influenced by birthdate (F_1,222_ = 59.64, *P* < 0.001) and BCI (F_1,219_ = 4.28, *P* = 0.009). In addition, it had a significant interaction between water and daytime temperatures (F_1,219_ = 6.56, *P* = 0.011), and water and nighttime temperatures (F_1,219_ = 4.77, *P* = 0.030), but no three-way interaction (*P* > 0.05). In summary, juvenile increase in tail width was improved by maternal water intake during gestation in cold nights or cold days (contrast = 0.003 ± 0.001, t = 2.53, *P* = 0.01) but water manipulation had no detectable effect in hot nights or hot days.

## Discussion

Understanding how animals respond to diel cycling temperatures and water availability can inform the development of more accurate predictive models for how physiological, behavioral, and life-history mechanisms might respond to future climate change scenarios (Rutshmann et al. 2024). These models can then help forecast the potential impacts on species distributions, population sizes, and community dynamics under different climate change projections. Ours is the first study to fill an important gap in our knowledge base by exploring the transgenerational effects of maternal hydration and temperature availability during gestation on offspring physiology, morphology, performance, and survival. Together with our previous study (Brusch et al. 2023), we found that long access to preferred temperature during the day confer benefits to both mothers and offspring, with some trade-offs in offspring traits. Conversely, high nighttime temperatures appear to impose challenges on mothers and also some offspring traits (reduced mass gain). We also found a negative impact of combined high day and nighttime temperatures on offspring survival. Additionally, water deprivation poses a challenge to homeostasis, but both mothers and offspring demonstrate resilience in coping with this stressor. Notably, the benefits of hotter environments are not always additive, highlighting the complexity of temperature-mediated effects on maternal and offspring outcomes.

### Higher day and nighttime temperatures

Previously, we found that an extended access to preferred temperature during daytime has several benefits for mothers, including increased food consumption, improved energetic state, and enhanced reproductive effort together with earlier births. Similarly, we found that higher nighttime temperatures advance gestation times but further challenge mothers through a lack of any dietary advantage and a reduction of the reproductive effort. These mothers were also more likely to have stillborn offspring and they had lower body conditions after giving birth. The much faster gestation times from higher temperatures meant that mothers were presumably at their phenological limit for embryonic development, since dual increase of nighttime and daytime temperatures did not accelerate gestation (Brusch et al. 2023). Earlier birth date is generally likely to benefit offspring in lizards (e.g., Le Henanff et al. 2013; Bodineau et al. 2024) and indeed we found that it improved postnatal growth rate during early life in outdoor enclosures. This, together with the age differences due to differences in birth dates, implied that offspring from late-breeding females were smaller on average after the summer growth season that those from early-breeding females. This growth advantage may benefit future survival, sexual maturation, and early life reproduction.

Here, we further found that the size of offspring (SVL) at birth was not influenced by any maternal treatments during gestation but the body condition (BCI) of neonates from mothers exposed to hot daytime temperatures was significantly lower compared to cold days. Body condition of newborn lizards is often determined by factors such as maternal investment, gestational conditions, and resource availability during early development. Newborn lizards with higher body condition also tend to have higher average survival rates compared to those in poorer body condition (this and previous study). Well-conditioned neonates are also better able to withstand environmental stressors, predation, and other challenges during the vulnerable early stages of life (Noble et al. 2018). In addition, neonate mass gain was lower in mothers exposed to hot nights when controlling for effects of birthdate, which suggests some negative carry-over effects of nighttime warming on offspring total mass but not tail width.

Newborn lizards are vulnerable to various environmental and physiological challenges. While their body condition can influence their ability to cope with these challenges, neonates are vulnerable to predation due to their small size, lack of defense mechanisms, and limited mobility, and are highly dependent upon food intake during their first days of life (Massot and Aragón 2013). To increase neonate survival, various strategies have evolved to avoid predators and forage more efficiently. In many species of lizards, including *Z. vivipara* (Le Galliard et al. 2004), newborns are known for their endurance running to avoid predators (e.g., green anoles [Loses 1990], skinks [Doody and Paull 2013], and leopard geckos [Landová et al. 2013]). Previous findings indicate that thermal condition during development and access to preferred temperature influences stamina at birth (Foucart et al. 2018). Endurance capacity is also important for thermoregulation efficiency and prey capture in active foragers like the common lizard. We found that offspring from mothers exposed to hot day or nighttime temperatures, but not both, had significantly lower endurance (Fig. 3). Reduced endurance in hot environments may make these offspring more vulnerable to predation and less efficient at foraging. However, mothers exposed to hot day or nighttime temperatures (but not both) had offspring with high survival rates in our outdoor enclosures, despite their lower endurance at birth (Fig. 5).

Survival rates were not influenced by birth date but decreased sharply in neonates from litters with combined hot nighttime and daytime temperatures, suggesting synergistic, negative warming effects on offspring viability. Higher temperatures in the day or night likely pushed mothers to their phenological limit, and these results might suggest that mother’s in higher whole-day temperatures did not have sufficient time to allocate appropriate resources to ensure the viability of their offspring. A mother’s ability to provision her offspring with adequate nutrients can have profound consequences for the offspring’s chances of survival in the early stages of life. As further evidence, we found that neonates from mothers exposed to hot day and hot nights had also significantly thinner tails (Fig. 1b). These impacts were even greater in neonates from mothers exposed to hot nights and deprived of water throughout gestation. The tail is an essential site for protein and lipid storage in many species of lizard, including *Z. vivipara*, and it might be especially important in neonates since newborn lizards have high metabolic demands as they grow and develop (Avery 1974; Batemen and Fleming 2009). They need to allocate energy towards processes like tissue growth, organ development, and thermoregulation. Lizards that hatch with larger fat reserves and better body condition might be better equipped to survive these challenges, as they have more energy stores to draw upon. More studies are needed to understand the mechanisms explaining the low survival of juveniles from the hot day and night treatment.

### Water deprivation

We previously found evidence that water restrictions increase the physiological costs of reproduction and challenge maternal homeostasis by increasing osmolality (i.e., dehydration), lowering body condition, and decreasing stored reserves (Brusch et al. 2023). Despite this, we found little effects of maternal water restriction on offspring viability and some positive effects on tail growth under specific thermal conditions. Earlier experiments had found complex effects of maternal water deprivation on postnatal growth and survival depending on offspring sex and postnatal environmental conditions (Lorenzon et al. 1999; Dupoué et al. 2018). Instead, our main findings suggest that dehydrated mothers may give birth to dehydrated offspring. Specifically, breathing rates were significantly higher in neonates from mothers exposed to hot days or nights (Fig. 2b) and heart rates were significantly faster in neonates from mothers held without water through gestation (Fig. 2c). Due to the small size of neonates, we did not collect blood samples to measure osmolality; however, these results suggest that the neonates were in fact dehydrated. Indeed, as a lizard becomes dehydrated, its blood volume decreases. This causes the heart to beat faster in an attempt to maintain adequate blood flow and oxygen delivery to tissues. Because total litter size and the SVL of neonates were not impacted by maternal treatments, this suggests that the lower BCI from water-deprived or high-temperature treatment mothers was partly explained by a reduced water investment during gestation, which led to signs of dehydration outlined above.

When the maternal environment provides a reliable predictor of the offspring’s future environment, maternal effects can facilitate the evolution of adaptive transgenerational plasticity, with offspring phenotypes better matching the expected environmental conditions (Shine and Downes 1999). Such anticipatory maternal effects have been demonstrated in some specific situations and appears beneficial on average in species with a more limited mobility (Wilson and Franklin 2002). It is thus reasonable to predict that mothers exposed to hot and dry conditions would give birth to offspring who are prepared for similar conditions, for example, with specific thermo-hydroregulation strategies adapted to warmer and more arid environments. On the contrary, we found that “dehydrated” offspring, from dehydrated mothers, also had higher standard TEWL rates at rest, a trait often associated with more mesic environments in reptiles (Lillywhite 2006). Transcutaneous evaporative water loss at rest occurs as water diffuses from the lizard’s body tissues and blood vessels through the skin and into the surrounding environment, and depends on surface and body shape, skin resistance to water loss, respiratory activity, and behavioral activity at rest as well as microclimatic conditions. These same lizards also had lower BCI and therefore probably higher surface-area-to-volume ratios, which may explain their higher water loss rates. Similarly, reducing blood flow is a physiological response to limit TEWL, even in dry-skinned reptiles (Dantzler and Bradshaw 2008). These dehydrated neonates, with their elevated heart rates, may have lost more mass over 12 hrs in controlled conditions because they were unable to reduce blood flow at rest. The other major factors that can influence TEWL in lizards include temperature, humidity, and activity level, and skin characteristics such as lipid composition (Lillywhite 2006). We controlled for the first three factors, which suggests that there may also be a difference in the structure or composition of dehydrated neonate skin that may influence its permeability and future studies should examine such a possibility.

## Author contributions

G.B., J.F.L.G., J.C., and O.L. conceived the ideas and designed methodology; G.B., R.B., R.S.B.G., and O.L. collected the data; G.B., J.F.L.G., and O.L. analyzed the data; and G.B., J.F.L.G., and O.L. led the writing of the manuscript. All authors contributed critically to the drafts and gave final approval for publication.

## Data Availability

The datasets supporting this article can be accessed at https://doi.org/10.5281/zenodo.14919971.

## Notes

From the symposium “Identifying the physiological mechanisms that underlie phenotypic responses to rapid environmental change” presented at the annual meeting of the Society for Integrative and Comparative Biology, January 3–6, 2024 Atlanta, Georgia.
